# Trophic level and proteobacteria abundance drive antibiotic resistance levels in fish from coastal New England

**DOI:** 10.1186/s42523-023-00236-w

**Published:** 2023-03-06

**Authors:** Benjamin J. Korry, Peter Belenky

**Affiliations:** Department of Molecular Microbiology and Immunology, Brown University, Providence, RI 02906 USA

## Abstract

**Background:**

The natural marine environment represents a vast reservoir of antimicrobial resistant bacteria. The wildlife that inhabits this environment plays an important role as the host to these bacteria and in the dissemination of resistance. The relationship between host diet, phylogeny, and trophic level and the microbiome/resistome in marine fish is not fully understood. To further explore this relationship, we utilize shotgun metagenomic sequencing to define the gastrointestinal tract microbiomes of seven different marine vertebrates collected in coastal New England waters.

**Results:**

We identify inter and intraspecies differences in the gut microbiota of these wild marine fish populations. Furthermore, we find an association between antibiotic resistance genes and host dietary guild, which suggests that higher trophic level organisms have a greater abundance of resistance genes. Additionally, we demonstrate that antibiotic resistance gene burden is positively correlated with Proteobacteria abundance in the microbiome. Lastly, we identify dietary signatures within the gut of these fish and find evidence of possible dietary selection for bacteria with specific carbohydrate utilization potential.

**Conclusions:**

This work establishes a link between host lifestyle/dietary guild, and microbiome composition and the abundance of antibiotic resistance genes within the gastrointestinal tract of marine organisms. We expand the current understanding of marine organism-associated microbial communities and their role as reservoirs of antimicrobial resistance genes.

**Supplementary Information:**

The online version contains supplementary material available at 10.1186/s42523-023-00236-w.

## Background

Fish are the most diverse group of vertebrates on earth with over 34,000 species inhabiting aquatic environments ranging from freshwater streams to the deep oceans [2]. They are essential to the ecosystems they inhabit, as well as the global food supply with fish providing over 3 billion people with 20% of their average protein consumption [3]. The global fishing industry is worth an estimated US$400 billion and employs nearly 60 million people worldwide making the health of the world’s fisheries of great economic importance [3, 4]. In order to ensure the future of this ecologically and environmentally invaluable group of organisms we must understand fish biology including their associated microbial communities. Fish harbor a large number of bacterial symbionts in their gastrointestinal tract (GIT), and these microbes have been shown to play a role in growth, development, and disease [5, 6]. They have coevolved with their microbial symbionts for over 400 million years [7], yet despite their antiquity and diversity, the microbiota of fish remain understudied compared to those of mammals. To date most studies have utilized 16S sequencing to understand how dietary supplementation impacts growth, development, and health in the context of the microbiome in commercial fish species raised in aquaculture [5, 6, 8–14]. These methods are limited to broad taxonomic changes in fish that are raised in captive settings. Less is known about the microbiota of wild fish populations and few studies have implemented shotgun sequencing technologies to gain a broader perspective on the functional gene content of fish microbiota [15].

The use of shotgun metagenomic sequencing on the gastrointestinal tract contents of wild fish populations provides not only the taxonomic structure of the gut microbiota, but it’s functional potential, detection of dietary signatures and parasites [16–18], and identification of antimicrobial resistance genes (ARGs). The gut microbiomes of several wild marine fish have been sequenced and data suggests that habitat, diet, and host phylogeny play a role in shaping the GIT microbiome [16, 19–25]. Previous studies identified *Proteobacteria*, *Firmicutes*, and *Bacteroidetes* as the major constituents of the marine fish GIT microbiome [6]. It has been suggested that marine fish have a host-specific microbiota composition [21, 26, 27], however work by Riiser et al. and others have shown that different ecotypes of the same species have divergent gut microbiota suggesting that environmental and dietary factors may be critical factors in determining the gut microbiome in some species [19–21]. Dietary specialization is associated with gut physiology in marine fish with herbivorous and omnivorous fish having longer gut length compared to carnivorous fish [6, 28]. These morphological differences across fish with different feeding habits are accompanied by observed increases in *Proteobacteria*, specifically *Vibrio* and *Photobacterium*, in some carnivorous species compared to herbivores and omnivores [6]. Further work has suggested that these carnivore-specific species, *Vibrio* and *Photobacterium*, may contribute to host digestion through production of enzymes [29].

Fewer studies have looked into ARGs harbored by fish associated microbes [25, 30, 31]. While the microbiome is of crucial importance to the host, studying gut microbiome associated ARGs is important for monitoring the dynamics of antimicrobial resistance in the marine environment and potential transmission to humans. A study of farmed saltwater fish identified an increased abundance of ARGs in farm effluent compared to the surrounding seawater [31]. Notably, *Vibrio* and *Photobacterium* were most commonly associated with ARGs [31]. Studies of the gut microbiota from deep sea fish revealed a surprising lack of ARGs considering the ubiquitous nature of resistance genes in the environment [25]. These few studies of antimicrobial resistance associated with marine fish highlight the need for improved research in this area.

Antimicrobial resistance is an increasing threat to human health with resistant organisms leading to 2.8 million infections and more than 35,000 deaths a year in the United States alone [32]. The natural environment is known to be a reservoir of ARGs with wildlife playing a role in the dissemination of resistant microbes [33]. Utilizing databases of ARGs [34–37] and traditional microbiology culturing techniques we are beginning to see that ARGs are widespread throughout different environments and organisms. Studies have found ARGs in humans [38–40], animals [41–44], soils [39, 45], caves [46], ice cores [47], and marine environments [43, 48, 49] demonstrating that resistance can be found wherever bacteria live. Understanding the role of marine environments as a reservoir of antimicrobial resistance is crucial due to widespread aquaculture and seafood consumption and resulting interactions between humans and marine bacteria.

Narragansett Bay is the largest estuary in New England and provides an essential habitat for numerous commercially and ecologically important species [50]. Demersal fish species that inhabit the bay, including *Peprilus triacanthus* (butterfish), *Stenotomus chrysops* (scup), *Paralichthys dentatus* (summer flounder), and *Mustelus canis* (smooth dogfish), each occupy different trophic positions based on previously defined dietary guilds—planktivore (butterfish), benthivore (scup), and predatory crustacivores/piscivores (summer flounder, smooth dogfish) [51]. Butterfish are found throughout the Atlantic coast of North America, occupying inshore habitats in the summer months and migrating offshore during cooler months [52]. This is a schooling species that feed primarily on planktonic prey including cnidarians, annelids, and other small prey found in the water column [52]. Scup are found along the North American Atlantic coast, forming schools that occupy inshore habitats in the summer and migrate offshore during winter [53]. These benthivores primarily consume amphipods and annelids [53]. Both butterfish and scup are also an important prey species for both the summer flounder and smooth dogfish [51, 54]. Summer flounder are a commercially significant flatfish that can be found throughout the Atlantic coast of North America [55]. They exhibit a similar seasonal migration to both the scup and butterfish, occupying shallow inshore waters during the summer months [55]. This species is primarily piscivorous, consuming fish, although stomach analyses show they also consume squid and crustaceans [51, 55]. Smooth dogfish are one of the most abundant shark species occupying the Western Atlantic along the North American coast, migrating from inshore habitats in the summer to offshore in the winter [54]. While primarily a crustacivore, this moderately sized shark also consumes fish as a part of its diet, including scup and butterfish [54]. These four species of interest represent a unique model in which to study the gut microbiome as they inhabit the same environment but differ in lifestyle and physiology, occupying specific trophic positions, and have direct predator/prey interactions.

The waters off the coast of New England are also important fisheries and are home to larger migratory predators including *Alopias vulpinus* (thresher shark), *Isurus oxyrinchus* (shortfin mako shark), and *Lamna nasus* (porbeagle shark). These large sharks reach lengths greater than 300 cm and are found in habitats around the globe. All three species are highly migratory [56], and are commonly found along the coast of New England where they prey primarily on fish—with threshers consuming primarily herring and mackerel [57], mako diets consists mainly of bluefish and mackerel [58], and porbeagle diets contains mackerel, herring, flatfish, groundfish, and smaller sharks including dogfish [59, 60]. Due to their position as apex predators, unique physiology, and highly migratory behavior, it remains a priority to better understand the shark GIT microbiome.

In this work we aim to use shotgun metagenomic sequencing to define the microbiome composition and relationship between host, microbiota, and antimicrobial resistance in the GIT of four demersal marine species as well as three large migratory shark species. We find inter and intra-species differences in the GIT microbiome based on host species and GIT sampling location, and that higher trophic level organisms with piscivorous diets have an increased abundance of ARGs. Additionally, this abundance of ARGs is positively correlated with the abundance of bacteria. Using a barcoding approach to identify non-host/bacterial DNA signatures in the shotgun sequencing data combined with a functional assessment of the microbiome, we are able to infer dietary habits and bacterial carbohydrate utilization. These habits play a role in determining the composition of the gut microbiota and, in turn, the levels of *Proteobacteria* and resulting abundance of detected ARGs.

## Results

### Sampling and collection

**Fig. 1:**
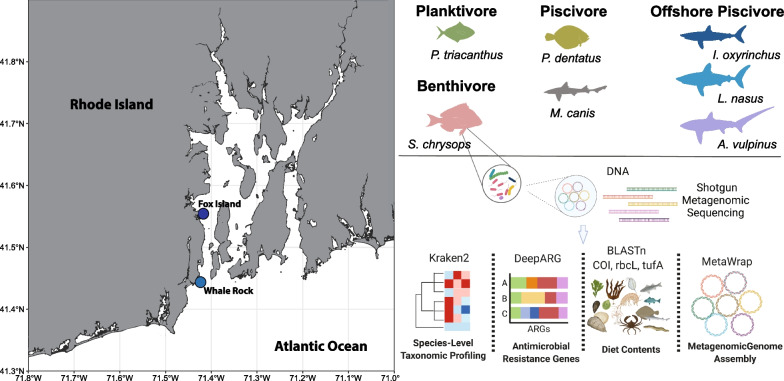
Sample Collection and Experimental Overview. Figure outlining the collection locations within the Narragansett Bay—Fox Island and Whale Rock. Trophic guild characterization of 7 fish and shark species examined in this study. Workflow of highlighting metagenomic analyses performed on DNA sequencing data

Sampling of four demersal fish species, butterfish, scup, summer flounder, and smooth dogfish, was performed using an otter trawl in Narragansett Bay, RI, USA (Fig. 1). Samples were collected at two sampling locations, Fox Island (upper bay) and Whale Rock (lower bay), during the months of May, June, July, August, and September between 2017 and 2021. During each sampling session, benthic water samples were also obtained using a Niskin flask. The spiral valve contents of three large offshore sharks, thresher, mako, and porbeagle sharks, were obtained from specimens caught for recreational shark tournaments in the offshore waters from Rhode Island to Maine during July 2018 and July 2019.

### Microbial diversity of the GIT of wild marine fish

**Fig. 2:**
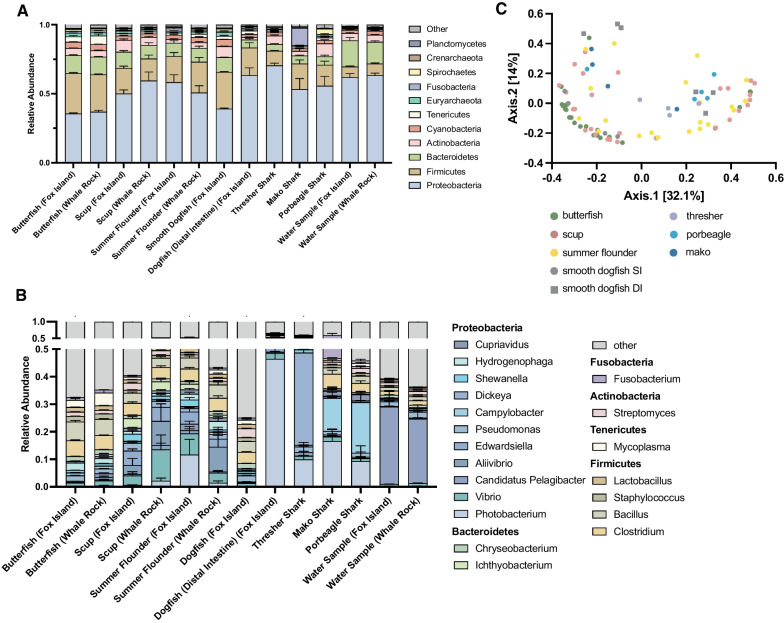
Taxonomy and Diversity of Fish and Environmental Samples. Relative abundance of bacterial phyla **A** and genera **B** averaged across samples within groups and error bars representing standard error of the mean. Principal coordinate analysis of Bray–Curtis Dissimilarity of all fish microbiota samples (excluding water samples) (**C**)

Utilizing a whole genome shotgun sequencing approach followed by read filtering and taxonomic assignment via the Kraken2/Braken pipelines we were able to define the microbiome composition of seven fish/shark species and their seawater environment. We find that the gut microbiota of all species is predominantly composed of *Proteobacteria* (50.4%), *Firmicutes* (20.8%), and *Bacteroidetes* (10.0%) (Fig. 2A).

This finding matches those of previous studies that have identified *Proteobacteria* and *Firmicutes* as being the major constituents of the gut microbiota of marine fish [5, 6, 19, 25, 61–64]. Within the phylum *Proteobacteria*, *Photobacterium* (5.6%), *Vibrio* (4.5%), *Alivibrio* (3.5%), and *Edwardsiella* (3.2%) were the most abundant genera found in the fish samples collected in the bay (Fig. 2B). Here we observe significantly increased levels of *Proteobacteria* in benthivorous/piscivorous (scup, summer flounder, smooth dogfish) species compared to a plankivorous species (butterfish) (Mann–Whitney U test, *p* < 0.0001). Previous studies have also identified an increased abundance in *Proteobacteria* in omnivorous and carnivorous organisms compared to herbivores [6, 21, 64, 65] suggesting trophic level and dietary guild play a role in the level of *Proteobacteria* present in the gut microbiota. Due to a high degree of variability within sample types, fish species did not group together significantly within a principal coordinate analysis of Bray–Curtis Dissimilarity (Fig. 2 C). However, the microbiomes of species clustered more separately when samples were separated by site and sampling time, suggesting that there may be significant temporal and spatial variability within the microbiome of fish (Additional file 1: Fig. S1 B, D). Between the samples collected at the two Narragansett Bay sampling locations there were notable differences in the microbiome composition of the summer flounder at the genus level; *Photobacterium* was significantly increased in the Fox Island population (padj < 0.05) and six less prominent genera were significantly increased in the Whale Rock population (padj < 0.05) (Additional file 1: Fig. S1 A, B; Additional file 2: Table S1; Additional file 3: Table S2). Surprisingly, no significant differences in taxonomy were found between the two sites in the butterfish and scup populations.

### Shark spiral valves harbor species-specific microbiota

**Fig. 3:**
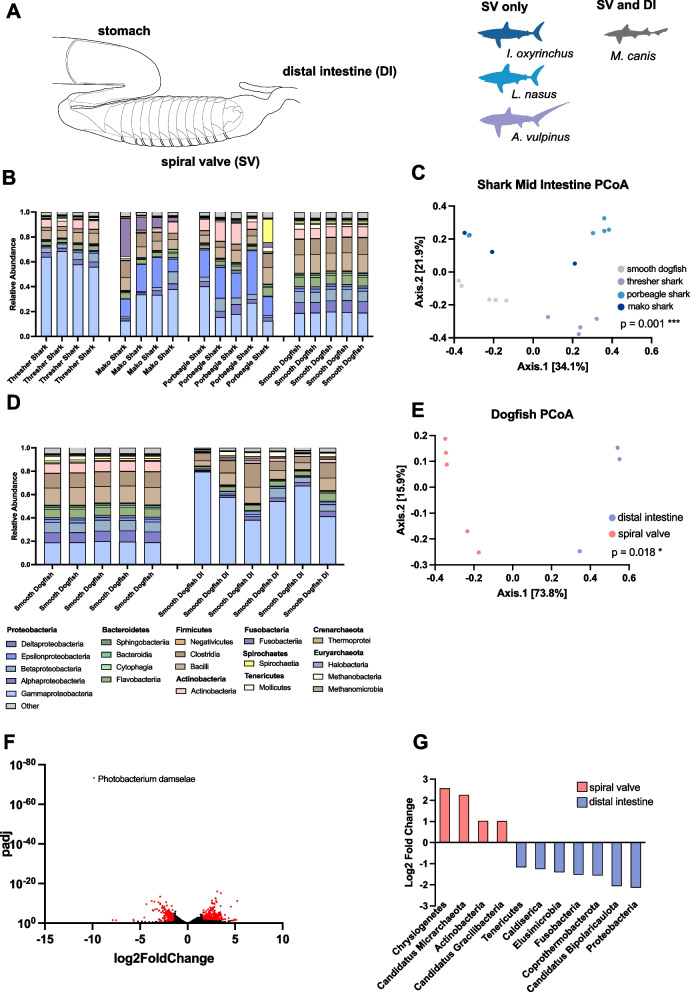
Taxonomy of Four Shark Species and Divergent Microbiota of Spiral Valve and Distal Intestine. Diagram of the elasmobranch GIT with the stomach, spiral valve, and distal intestine labeled (adapted from De luliis and Pulerà 2019) [1], and list of the four shark species included in this study (**A**). Relative abundance of bacterial classes across individual spiral valve contents from four shark species (**B**). Principal coordinate analysis of Bray–Curtis Dissimilarity of spiral valve microbiota cluster by species and are significantly different from one another (PERMANOVA, *p* = 0.001) (**C**). Relative abundance of bacterial classes across individual spiral valve and distal intestine contents isolated from smooth dogfish (**D**). Principal coordinate analysis of Bray–Curtis Dissimilarity of smooth dogfish spiral valve and distal intestine microbiota cluster by GIT location and are significantly different from one another (PERMANOVA, *p* = 0.018) (**E**). Volcano plot of differentially abundant species between the smooth dogfish spiral valve and distal intestine. Points in red represent significantly different species with an adjusted *p*-value of < 0.05 and log2 fold change of > 1.5 (**F**). Significantly differentially abundant phlya with an adjusted *p*-value of < 0.05 and log2 fold change of > 1.5. Phyla more abundant in the spiral valve are shown in red and those in more abundant in the distal intestine are blue (**G**)

The gut microbiota of sharks has only been characterized in a few reports [22, 23, 62, 66, 67], and represents an understudied area of shark physiology, which likely plays a major factor in host health. Here, we define the microbiota of four shark species, the mako shark, thresher shark, porbeagle, and smooth dogfish. Sharks have unique digestive architecture defined by the spiral valve, an organ that maximizes absorption and minimizes the length of GIT by increasing surface area through a corkscrew-like arrangement of intestinal tissue (Fig. 3A).

The spiral valve of all sharks was dominated by *Proteobacteria* (53.9%) and *Firmicutes* (18.0%), with *Photobacterium* (17.5%), *Campylobacter* (6.0%), and *Dickeya* (5.7%) the most prominent genera (Fig. 3B, 2B). Analysis of the Bray–Curtis Dissimilarity metric revealed a significant difference between the microbiota of each species (PERMANOVA, *p* = 0.001), defined by distinct clustering in a principal coordinate analysis (Fig. 3C). Previous studies of Elasmobranchii have also found an abundance of *Photobacterium* as well as *Campylobacter* in the spiral intestine of sharks [62, 67], but to date only one study utilizing 16S sequencing has examined the taxonomic differences between regions of the shark GIT [66].

Here, we compare the microbiota of the spiral valve (SV) to the distal intestine (DI) in smooth dogfish. The principal coordinate analysis plot of the Bray–Curtis Dissimilarity metric displays the significantly distinct clustering of the SV and DI microbial communities (PERMANOVA, *p* = 0.018) (Fig. 3E). These disparate communities are defined by a significantly greater abundance of *Proteobacteria* in the DI (63.3%) compared to the SV (39.1%) (*p* = 4.55E-05), and a significantly reduced abundance of Actinobacteria in the DI (1.8%) compared to the SV (8.0%) (*p* = 1.89E-08) (Fig. 3D, F, 3G). The most differentially abundant species between GIT sites was *Photobacterium damselae*, which was significantly more abundant in the DI compared to the SV (log2FC = 9.84, padj = 4.52E-74) (Fig. 3E). Such differences in microbial composition were not found in the GIT of the previously studied bonnethead shark (*Sphyrna tiburo*) [66], suggesting this may not be a universal phenomenon among sharks.

### The GIT microbiota of marine fish act as a reservoir of ARGs which are associated with proteobacteria

**Fig. 4:**
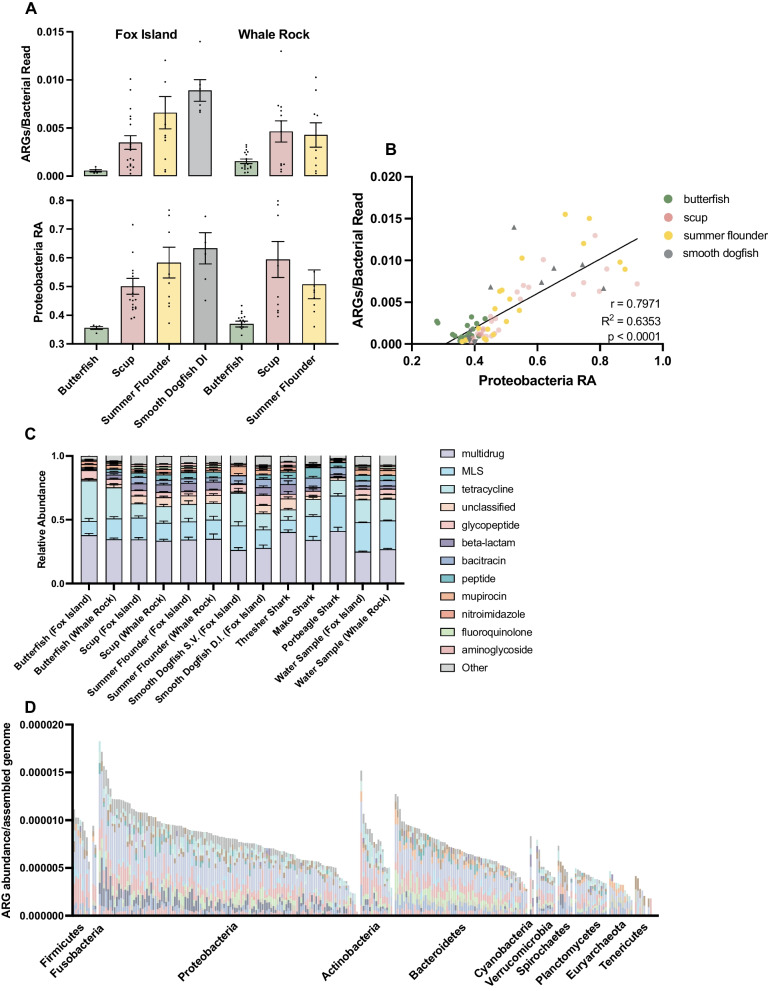
Antimicrobial Resistance and Association Between ARGs and *Proteobacteria.* (Bottom) Relative abundance of *Proteobacteria* in each species at the Fox Island (left) and Whale Rock location (right) averaged across samples with error bars representing the standard error of the mean (**A**). (top) ARGs normalized to bacterial reads in each species at the Fox Island (left) and Whale Rock location (right) with bars representing mean ± standard error of the mean (**A**). The bars in A (top) correspond to those in A (bottom). Correlation between ARGs (y-axis) and *Proteobacteria* relative abundance (x-axis) (r = 0.7971, R^2^ = 0.6353, *p* < 0.0001) (**B**). Relative abundance of ARG classes averaged across samples for each species at each location with error bars representing standard error of the mean (**C**). Abundance of ARGs in each MAG with 50% ≥ completeness and ≤ 5% contamination (**D**). The colors of the stacked bars in this plot correspond to those in the legend above

Environmental reservoirs of antimicrobial resistance play an important role in the selection, proliferation, and transfer of resistance genes [41]. We used the computation tool DeepARG [68] to identify resistance genes and find that the gut microbiota of marine fish represent one such reservoir of ARGs. Across all fish GIT samples we detected 518 different resistance genes covering 27 antibiotic resistance classes (Additional file 4: Table S3). The most abundant resistance gene classes were multidrug (34.3%), macrolide, lincosamide, streptogramin (MLS) (16.1%), tetracycline (16.0%), and beta-lactam (4.6%) (Fig. 4C). Recently, Collins et al. found multidrug and beta-lactam resistance genes in the microbiota of deep-sea fish [69], and similarly a study of ocean waters around the globe found tetracycline, beta-lactam, and multidrug resistance genes to be the most prevalent resistance gene types in seawater [70]. These findings suggest resistance mechanisms may be conserved across bacteria that inhabit the marine environment and fish GIT.

An increase in antibiotic resistance gene abundance was associated with certain fish/shark species, specifically in higher trophic level organisms (Fig. 4A, top). In general, those fish that exhibited piscivorous feeding behavior, occupying a higher trophic level, had a greater burden of antibiotic resistance. Rowan-Nash et al. found a significant correlation between *Gammaproteobacteria* and ARGs in human gut microbiota samples suggesting that the presence of certain bacteria may be driving levels of resistance in host-associated microbial communities [40]. Expanding on this idea, we examined the relationship between ARGs and *Proteobacteria* in the GITs of fish and found that samples from piscivores with a higher relative abundance of *Proteobacteria* harbored an increased abundance of ARGs compared to planktivorous/benthivorous species with less *Proteobacteria* (Fig. 4A, bottom, Additional file 1: Fig. S2). A correlation analysis between ARG abundance and *Proteobacteria* relative abundance in fish within Narragansett Bay showed a significant positive correlation (r = 0.7971, R^2^ = 0.6353, *p* < 0.0001, Pearson’s correlation) (Fig. 4B). When we factored in the large offshore shark species, we find that this trend generally holds true with the exception of the thresher shark, which despite having high levels of *Proteobacteria* had relatively low levels of ARGs (Additional file 1: Fig. S3). These findings show that fish with high levels of *Proteobacteria* are likely to have an increased level of detectable ARGs. Furthermore, this may suggest that higher trophic level organisms with a more carnivorous diet and *Proteobacteria* rich gut microbiota will have a greater resistance gene burden.

In order to determine the bacterial hosts of these resistance genes, metagenomically assembled genomes (MAGs) were assembled from cleaned sequencing reads using the MetaWRAP assembly pipeline [71] and assemblies were subsequently queried for ARGs. From all metagenomic reads across fish and water samples, we assembled 267 MAGs covering 9 bacterial phyla (Additional file 1: Fig. S4). We found that the MAGs from *Firmicutes* (*n* = 8), *Fusobacteria* (*n* = 2), and *Proteobacteria* (*n* = 121) had the highest prevalence of ARGs, and had significantly more resistance genes than MAGs from *Bacteroidetes*, *Verrucomicrobia*, *Spirochaetes*, *Planctomycetes*, and *Tenericutes* (*n* = 104) (Mann–Whitney U test, *p* < 0.05) (Fig. 4D). Notably, the second most ARG-rich MAG was identified as *Photobacterium damselae*, which occurred at a high abundance in all the piscivorous fish and shark gut microbiota supporting the theory that higher trophic level organisms may harbor more ARGs (Fig. 2C).

### Inferring diet through metabarcoding of GIT shotgun metagenomic data

Traditionally techniques to study diet in wild animals, such as direct observation or stomach content analysis, have been low throughput and time consuming and are unable to identify phenotypically indistinguishable or rapidly digested prey items [72]. The use of DNA-barcoding methods circumvents these issues by providing molecular level resolution that reduces the need for human identification of physical dietary components [72]. Here, we utilize DNA-metabarcoding targeting the cytochrome c oxidase subunit I (COI) [73], elongation factor TU (*tufA*), and ribulose-1,5-bisphosphate carboxylase (*rbcL*) genes [74] to identify the diet and potential GIT parasites of seven marine species.

**Fig. 5:**
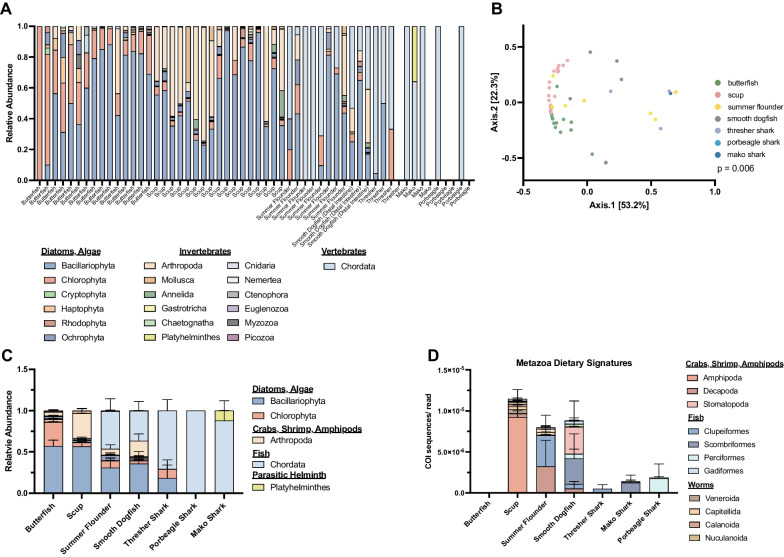
Dietary Signature Identification Through GIT Metabarcoding. Relative abundance of dietary components at the phylum level determined through metabarcoding of shotgun metagenomics using the *tufA*, *rbcL*, and *COI* genes (**A**). Principal coordinate analysis presenting the Bray–Curtis Dissimilarity analysis of the dietary components of seven fish species. Each species’ diet profile grouped separately (PERMANOVA, *p* = 0.006) (**B**). Relative abundance of dietary components at the phylum level averaged across samples with error bars representing standard error of the mean (**C**). Normalized abundance of Metazoan dietary signatures determined by using the *COI* reads from shotgun metagenomic sequencing of the fish GIT contents (**D**). Bars represent the average across samples within species and error bars represent standard error of the mean (D). (All data here are from the 69 samples collected in 2021 and do not include previous collections)

Of the seven species examined in this study, four occupy a shared demersal habitat in Narragansett Bay, RI providing an opportunity to detect interspecies predation and differential dietary preferences within a habitat. The planktivorous butterfish had a diet primarily consisting of diatoms (Bacillariophyta), algae (Chlorphyta, Ochrophya, Haptophyta), and to a lesser extent arthropods (Arthropoda), characteristic of an organism occupying a low trophic level (Fig. 5A, C). The benthivorous scup occupies a higher trophic level than the butterfish, characterized by dietary signatures of diatoms (Bacillariophyta), arthropods (Arthropoda), and segmented worms (Annelida) which were known to be a major prey source for this benthic species (Fig. 5A, C) [75]. At the order level we find that the Metazoan portion of the scup diet is derived from amphipods (Fig. 5D). Previous dietary studies of both the summer flounder and smooth dogfish in New England waters identified these species as high trophic level predators preying on fish, squid, and crabs [51, 54]. It is notable that due to the feeding patterns of these species they were sometimes captured with empty stomachs and intestinal tracts resulting in an absence of detectable DNA markers making diet identification impossible (Fig. 5A). We find that these highly carnivorous species prey primarily on chordates in the class Actinopterygii (ray-finned fishes) as well as arthropods (Fig. 5A, C, D). In summer flounder the Metazoan derived diet came from primarily Decapoda (crustaceans) and Clupeiformes (herring and anchovy family) (Fig. 5D). The smooth dogfish DI contained Metazoan signatures of Stromatopoda (mantis shrimp) and fish across several orders (Fig. 5E). DNA markers corresponding to butterfish and scup were found in the GIT of the high trophic level predators (summer flounder and smooth dogfish) suggesting that predation occurs within this benthic food web and represents a possible route of bacterial and ARG transfer from lower- to higher-trophic level organisms. We also obtained dietary signatures from three large migratory shark species that play an important role in the food web as apex predators. All three sharks exhibited piscivorous diets based on metabarcoding (Fig. 5A, C, D). A closer look at order level taxonomy revealed that each shark had a fairly specialized diet with DNA from only one or two different prey species (Fig. 5D). The COI dietary signatures for the thresher, mako, and porbeagle sharks were primary from Clupeiformes, Scombriformes, and Perciformes, respectively (Fig. 5D). Using this metabarcoding approach for dietary contents we confirmed that the summer flounder, smooth dogfish, mako, thresher, and porbeagle sharks all had highly piscivorous diets compared to the butterfish and scup. Furthermore, each species harbored a significantly distinct diet that was host specific (PERMANOVA, *p* = 0.006) (Fig. 5B). These trends in prey preference likely influence the microbial communities inhabiting the GIT as diet is a strong modulator of the microbiome. From a metabarcoding analysis of wild marine fish GIT samples we were able to infer diet, trophic interactions, and gain insights into the role of host diet in shaping the microbiota through nutrient availability and potential bacterial transfer between diet and host.

### Functional differences in the microbiota linked to host diet and trophic level

**Fig. 6:**
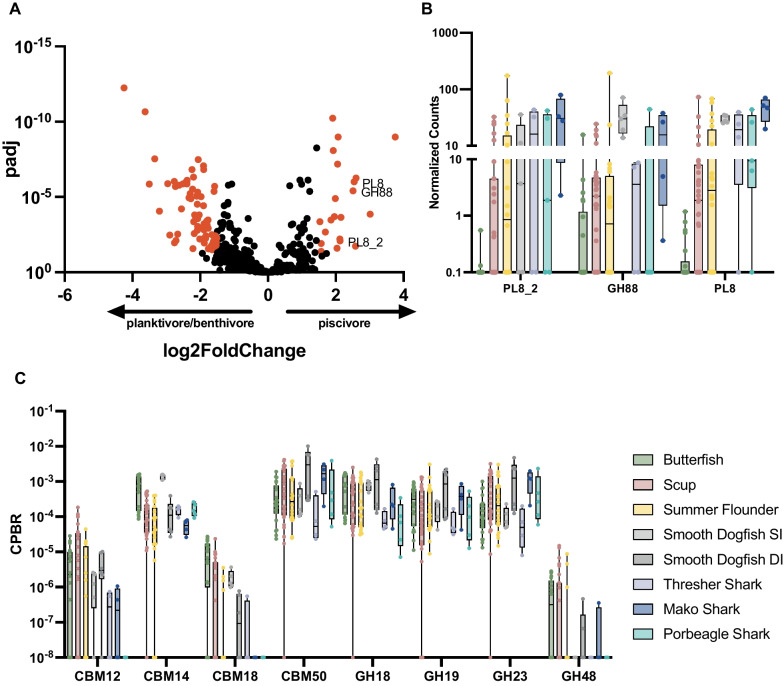
Functional Differences in the Microbiota Linked to Host Diet and Trophic Level. Volcano plot of differentially abundant CAZymes between planktivores/benthivores (butterfish and scup) and piscivores (summer flounder, smooth dogfish, thresher shark, porbeagle shark, mako shark) with three chondroitin metabolism genes highlighted (**A**). Points in red represent significantly different species with an adjusted *p*-value of < 0.05 and log2 fold change of > 1.5. Normalized counts of three chondroitin metabolism genes in each sample type (**B**). Normalized counts of eight CAZymes related to chitin metabolism across all samples (CPBR represents Copies per Bacterial Read) (**C**)

The gut microbiota plays an important role in host digestion, increasing nutrient availability and uptake [5, 21, 76]. Investigation into the carbohydrate-active enzymes (CAZymes) known to play a role in metabolism of dietary polysaccharides revealed 120 differentially abundant CAZymes between the piscivorous and planktivorous/benthivorous species suggesting that the divergent diets of these groups may have an impact on the functional capacity of the microbiome (Fig. 6A).

We find that several CAZymes linked to chondroitin metabolism are significantly enriched in the piscivores compared to planktivores/benthivores (log2fc > 1.5, padj < 0.05) (Fig. 6A, B). Interestingly, these genes were predominantly detected in MAGs isolated from the piscivorous species, summer flounder, smooth dogfish, thresher, mako, and porbeagle sharks (Additional file 5: Table S4). Chitin is one of the most abundant polysaccharides in nature and makes up the exoskeletons of many arthropods [77, 78]. Several chitinases were detected across nearly all the gut microbiota samples collected (Fig. 6C).

## Discussion

Studying the microbiota of wild marine fish is important for monitoring the health of populations, understanding fundamental fish biology, and evaluating their role as an environmental reservoir of antimicrobial resistance. Here, we utilize shotgun metagenomic sequencing to define the microbial taxonomic composition, ARG burden, and dietary DNA signatures from GIT samples of seven marine fish/sharks. Across all GIT microbiota samples, we find a predominance of *Proteobacteria* and *Firmicutes*, which is consistent with previous reports of marine fish gut microbiota [5, 6]. Each species harbored unique taxonomic profiles, which remained consistent between two sampling locations within the bay. The exception was that of the summer flounder, which had seven differentially abundant genera between the two sites including a significant increase in *Photobacterium* in the Fox Island samples (Fig. 2A, Additional file 1: Fig. S1). This suggests that the microbiota of summer flounder may have characteristics unique to either the upper or lower bay locations, while the butterfish and scup populations are more homogeneous. This is particularly interesting given that previous research of summer flounder within Narragansett Bay has found sex-based differences between the two sampling locations. Data suggests that the inshore habitat (Fox Island) has a higher proportion of females during the months our collections took place (May—September), whereas lower in the bay the Whale Rock location has a lower proportion of females [79]. Combined with other work showing that female summer flounder exhibit a faster growth rate [80], and that sex has an effect on microbiome composition in fish [81], future studies could examine whether the changes in summer flounder microbiota observed between the two sites are sex dependent. While the butterfish and smooth dogfish appeared to have consistent microbiome profiles across individuals, the scup and summer flounder samples appeared to have greater intra-species microbiome variability highlighted by samples with high abundances of *Proteobacteria* (Additional file 1: Fig. S2). It is unclear the driving factors behind this variability, nor is it unique to our data set [81], but it should be taken into consideration when evaluating the data shown here and in future studies of fish microbiota.

The GIT microbiota of sharks has been studied in only a few species to date [22–24, 62, 66, 67], despite their important role as apex predators within the marine trophic structure. We define the microbiota of four shark species including three highly migratory pelagic species. These organisms shared core bacterial taxa at the class level (Fig. 3B), while still having species- specific microbiome profiles (Fig. 3C). The universal presence of *Photobacterium* across all shark samples presented here, as well as previously published shark GIT microbiomes [62, 67] suggests that this genus is an essential part of the microbiota in these animals. Interestingly, *Campylobacter* seems to be a significant member of the GIT microbiota of sharks in the Lamnidae family, of which representative species from all three extant members of this family (*Carcharodon* [67], *Isurus* [this study], *Lamna* [this study]) have shown an abundance of *Campylobacter* (Fig. 2B). This is in contrast to sharks across almost all other Elasmobranchii families (Triakidae [this study], Alopiidae [this study], Carcharhinidae [22, 67], Rhincodontidae [22, 67], Sphyrnidae [23, 66], Ginglymostomatidae [22]) that did not report significant levels of this bacterial genus in their GIT, suggesting that this may be evidence of phylosymbiosis [82], though more work would be needed to substantiate this theory. Additionally, we observe significant differences between the SV and DI within smooth dogfish specimens (Fig. 3D, E, F, G). This is in contrast to 16S sequencing studies of the bonnethead shark which observed no differences between these two sites along the GIT [66], thus providing novel evidence that there may be spatial differentiation of the microbiota within the elasmobranch GIT. The SV and DI may represent unique ecological niches for commensal microbes, and that perhaps nutrient availability, host immunity, or oxygen levels may act as selective factors for bacterial colonization in these regions of the smooth dogfish GIT. Our finding suggests that sampling method (cloacal swab vs direct sampling of spiral valve contents) and location along the GIT have significant impact on the detected microbiome profile.

The position of *Proteobacteria* as a commensal in the microbiome of marine organisms is well established. Our data is consistent with this finding, and shows that the genus *Photobacterium* is associated with piscivorous fish/sharks. While *Photobacterium damselae* is known to cause pathogenesis in both fish and humans [83, 84], ours and several other studies have recently found it in the GIT microbiome of marine fish suggesting it is likely a member of the natural gut flora [62, 67]. The *Photobacterium* MAGs assembled from both summer flounder and smooth dogfish contained genetic regions assigned to four different CAZymes related to chitinase activity (CMB50, GH18, GH19, and GH23) (Additional file 5: Table S4) [85]. Due to the abundance of chitinous prey sources (Arthropoda) identified in the gut of many of these samples, perhaps commensal *Photobacterium* play a role in the utilization of dietary derived chitin. Future studies should focus on strain level analyses of *Photobacterium* to characterize potential genomic and phenotypic differences between pathogenic and commensal strains.

Environmental microbiomes act as reservoirs of bacteria harboring antimicrobial resistance. Here, we define the resistome of seven wild marine fish/shark species and find that multidrug, MLS, tetracycline, and beta-lactam resistance genes are prevalent among these bacteria (Fig. 4C). Interestingly, we identify a positive association between the abundance of *Proteobacteria* and level of ARGs within fish GIT microbiomes (Fig. 4B). *Proteobacteria* abundance was found to be higher in piscivorous species compared to planktivores, likely leading to a greater ARG burden in higher trophic level species (Additional file 1: Fig. S3B). This study represents the first known report linking trophic level to ARG abundance. These findings are critical to understanding the dynamics of resistance in the context of marine food webs as well as the prevalence of resistant bacteria (especially pathogens) in highly migratory species such as the mako, thresher, and porbeagle sharks which have the ability to disperse such bacteria across great distances [86]. A recent study by Collins et al. identified a sparsity of resistance genes in the GIT of deep-sea fish that presumably experience a low level of anthropogenic impacts compared to the coastal species presented here [25]. The proximity to humans could be one factor leading to the much greater number of ARGs recovered from the samples presented here compared to those collected in the deep-sea. Previous work has shown that marine sediments with greater proximity to human activity have significantly higher abundances of ARGs compared to those in the less anthropogenically impacted waters [49, 87, 88].

The composition of the microbiome is greatly affected by host diet, though interactions between diet and microbiota composition in wild fish populations remain less well understood. Here we utilize a metabarcoding approach to identify non-host/bacterial DNA in the GIT of marine fish and detect dietary signatures and potential host parasites. Using several marker genes, we were able to discern prey items from GIT contents, representing a potentially less invasive alternative to traditional stomach content analyses. Interestingly, while squid has been reported to be a significant portion of the diet of summer flounder and smooth dogfish [51, 54], we did not observe any dietary signatures indicative of the longfin squid native to Narragansett Bay. This could be due to the rapid degradation of this type of prey item in the stomach, and if so, would be an important caveat to using this approach for diet detection. The fact that dietary signatures could not be discerned for all samples may be a factor of gut transit time and that only a single feeding period is detected at one time using these metabarcoding techniques. While this is a possible limitation, this short time frame of detection is also a strength as it provides a snapshot of recent dietary activity. Additionally, we were able to identify a known parasitic Platyhelminthes worm of the genus *Clistobothrium* in the spiral valve contents of one of the mako shark specimens [89]. Metagenomic assembly allowed us to assemble a full COI sequence for this parasite displaying the power of shotgun metagenomic sequencing in identification of GIT parasites in wild animals (Additional file 1: Fig. S5). Overall, the use of molecular barcoding techniques from shotgun metagenomic data provided insights into host dietary habits and trophic interactions between species in a complex marine ecosystem. This information is valuable to understanding the nutrient availability driving microbial selection within the GIT and ultimately shaping the gut microbiome.

Host diet in turn plays a key role in determining the makeup of the gut microbiome, and evidence shows that dietary modulation and macronutrient availability can drastically alter the composition and function of the intestinal flora [13, 27, 90]. Through previous stomach content analyses [51, 54], and our own metabarcoding analysis (Fig. 5), we are able to gain an understanding into the role of diet in shaping the gut microbiome of these marine fish. Glycosaminoglycans, including chondroitin, are a group of diverse polysaccharides that are components of a variety of tissues including cartilage derived from mammals, marine fish, squid, and other organisms [91–96]. The diet of piscivorous fish, such as those studied here, include a number of organisms known to contain chondroitin (Arthropoda and Chordata). Thus, the piscivorous fish and sharks occupying a higher trophic level would be expected to have greater dietary intake of this polysaccharide compared to the butterfish and scup, whose prey is less rich in chondroitin. The results of our analysis showed CAZymes associated with chondroitin metabolism are significantly higher in piscivores compared to planktivores/benthivores (Fig. 6A, B). This data suggests that host diet, associated with trophic level and dietary guild, may select for bacteria with particular carbohydrate utilization patterns. In this case, piscivorous fish and sharks likely have a more chondroitin rich diet and the abundance of chondroitin could provide an ecological niche for bacteria with chondroitin lyase and hydrolase enzymes. In addition, the presence of chitinases across all samples in our CAZyme analyses suggests that the ability to utilize chitin may be a widespread trait among marine associated microbiomes likely due to the fact that chitin is ubiquitous in this environment. Our evaluation of carbohydrate active enzymes within the fish gut microbiota suggests that the availability of dietary polysaccharides associated with different trophic levels may have a role in selecting for certain bacteria based on polysaccharide utilization. This finding has the potential to link host trophic level and related prey consumption with selection for specific microbes.

While this study effectively uses molecular techniques to define the microbiome, resistome, and dietary signatures, there are several limitations. As with any study assigning taxonomy or gene identifications to sequencing data, the results are limited by the completeness of existing databases. This is evidenced here by the fact that, in some cases, a high proportion of the host-filtered reads (up to 90%) remained unclassified after taxonomic assignment. Further characterization of microbes from understudied environments is needed to improve databases in order to better characterize these unique microbial communities. As well as taxonomic assignment, our ability to identify antibiotic resistance genes is limited by the available sequence databases. For an ARG to be present in a database, it must be previously characterized. The characterization of resistance disproportionately occurs in pathogens due to the importance of resistance in clinical microbiology samples, and many human pathogens are *Proteobacteria*. Thus, there is a potential for existing ARG databases to be biased towards *Proteobacteria*l ARGs, a fact that must be examined further to obtain a true picture of resistomes. Additionally, the data presented here is derived from shotgun DNA sequencing, thus it is only able to infer the functional potential of the genes identified. Without RNA sequencing and proteomics, we are unable to make strong conclusions regarding the activity of the microbes that inhabit the GIT. While these microbiome samples represent unique, previously unstudied species, there were limitations in obtaining more samples and thus it is possible some microbiome differences were not observed due to a low number of individuals sampled from each species. Despite these limitations, we are able to provide valuable insights into the microbiota and resistomes of wild marine fish occupying diverse dietary guilds and ecological niches.

## Materials and methods

### Sample collection

All Narragansett Bay fish samples, butterfish (*n* = 22), scup (*n* = 31), summer flounder (*n* = 20), smooth dogfish spiral valve (*n* = 5), smooth dogfish distal intestine (*n* = 6), were collected in the months of May, June, July, August, and September during 2017—2021 from the fish trawl surveys conducted by the University of Rhode Island Graduate School of Oceanography. Specimens were collected according to the IACUC protocols covering both this study as well as the work of the collection vessel. Fish trawl for samples was approved and permits were obtained from the Rhode Island Department of Environmental Management. The trawl was conducted by the R/V *Cap’*n* Bert* which utilized an otter trawl net with an effective opening of 6.5 m and towed at 2 knots for 30 min. Trawling was performed at two sites in Narragansett Bay, Rhode Island: Fox Island and Whale Rock (Fig. 1). After the trawl was emptied on the deck, living target fish were humanely euthanized via a blunt force blow to the head followed by pithing (as recommended by the 2020 AVMA guidelines for euthanasia). Following euthanasia, fish were dissected and the intestinal contents were emptied into Zymo Research bashing bead lysis tubes (Irvine, CA, USA) containing 750uL of ZymoBIOMICS Lysis Solution (Irvine, CA, USA), shaken, and stored on ice until extraction. Water samples were collected ~ 1 m above the seafloor using a Niskin flask. For each individual seawater sample (*n* = 12), one liter of seawater was filtered through a 0.22 μm membrane from which a 3 × 3 cm section was added to a Zymo Research bashing bead lysis tubes (Irvine, CA, USA) containing 750uL of ZymoBIOMICS Lysis Solution (Irvine, CA, USA), shaken, and stored on ice until extraction. The three large offshore shark species, thresher (*n* = 4), mako (*n* = 4), and porbeagle shark (*n* = 5), were collected from specimens caught as part of recreational shark tournaments in Massachusetts and Rhode Island. The samples were caught in the offshore waters from Rhode Island to Maine. All shark samples were collected postmortem from sharks collected by licensed recreational fishermen. The sharks were dissected and contents from the spiral valve were transferred into Zymo Research bashing bead lysis tubes (Irvine, CA, USA) containing 750uL of ZymoBIOMICS Lysis Solution (Irvine, CA, USA), shaken, and stored on ice until they could be frozen and subsequently extracted.

### DNA extraction

DNA was extracted with the ZymoBIOMICS DNA miniprep kit from Zymo Research (Irvine, CA, USA) following the manufacturer’s instructions, with final elution in 100 μl of molecular grade H2O. Extracted DNA was quantified using a Qubit™ 3.0 Fluorometer (Thermo Fisher Scientific, Waltham, MA, United States).

### Library preparation and sequencing

Metagenomic libraries for samples BK001—BK072 were prepared using the NEBNext Ultra™ II FS DNA Library Prep Kit for Illumina (NEB) (Ipswich, MA, USA) and libraries were sequenced on the NovaSeq 6000 with v1.5 reagents. Metagenomic libraries for samples BK073—BK114 were prepared using the iGenomeX Riptide High Throughput Rapid DNA Library Prep (Twist Bioscience, San Francisco, CA, United States).

### Metagenomic analysis

#### Read processing and filtering

Raw reads from metagenomic sequencing were processed using the KneadData wrapper script [97]. Reads were then trimmed using Trimmomatic (version 0.36) with SLIDINGWINDOW set at 4:20, MINLEN set at 50, and ILLUMINACLIP: TruSeq3-PE.fa:2:20:10 [98]. Sequences from contaminating host were filtered out using Bowtie2 [99]. Since fully sequenced genomes of the host species used in this study have not yet been sequenced, the next most phylogenetically similar fish with sequenced genomes were used as a reference during read filtering; *Paralichthys olivaceus* (PRJNA344006), *Spondyliosoma cantharus* (PRJEB12469), *Pampus argenteus* (PRJNA240272), *Scyliorhinus canicular* (PRJEB35945), and *Carcharodon carcharias* (PRJNA725502). In addition to this preprocessing, bacterial ribosomal reads were removed from the datasets using the SILVA 128 database [100].

#### Taxonomic Identification

Taxonomic classification of metagenomic reads was performed using Kraken2 (version 2.1.2) [101]. The taxonomic output was analyzed in R (version 4.1.2) using the phyloseq package (version 1.38.0) to calculate alpha and beta diversity [102]. The PCoA analysis was performed using the Bray–Curtis dissimilarity metric [103].

#### Identification of antimicrobial resistance genes

Processed reads were joined using the fastq-join function of the ea-utils package [104] and queried for antibiotic resistance genes using DeepARG (version 2) [68] using the default settings (0.8 minimum coverage of alignment, E-value cutoff 1e-10, 50% minimum percentage of identity). Assembled genomes were queried for resistance genes using DeepARG (version 2) using the –genes flag and the default settings (0.8 minimum coverage of alignment, E-value cutoff 1e-10, 50% minimum percentage of identity).

#### Identification of functional genes

Additionally, using the SAMSA2 pipeline [105] clean reads were merged using Paired-End Read Merger (PEAR) (version 0.9.10) [106] and aligned to the RefSeq, CAZy, and SEED subsystems databases using DIAMOND (version 0.9.12) [85, 105, 107–109].

#### Metabarcoding for diet detection

The origins of non -host/bacterial DNA content in the gut was determined by using BLASTN to align cleaned, merged reads to a custom database containing the cytochrome C oxidase subunit I gene (COI) sequences in the database generated by the CO-ARBitrator algorithm developed by Heller et al. [73] and all unique sequences from NCBI Gene search of the genes *tufA*, encoding for elongation factor TU, and *rbcL*, encoding for ribulose-1,5-bisphosphate carboxylase. Non host/bacterial DNA content alignments were filtered based on alignment length of ≥ 100 bp and percent identity ≥ 97% with any singletons removed.

#### Metagenomic assembly, binning, and taxonomic identification

Metagenomic assembly was conducted using the metaWRAP pipeline [71] with the –megahit flag. Binning was conducted using the metaWRAP binning module employing metabat2, maxbin2, and CONCOCT binning software. Final bins with completion ≥ 50% completeness and < 5% contamination were used for downstream analysis. The Bin Annotation Tool (BAT) (version 5.2.3) was used for taxonomic classification of metagenome-assembled genomes [110].

#### Generation of phylogenetic tree

The phylogenetic tree of MAGs was generated using PhyloPhlAn 3.0 using the “–diversity high” flag [111]. Node labels were based on the lowest taxonomic assignment of the Bin Annotation Tool.

#### Statistical analyses and figure generation

Differential abundance of sequence annotations was determined using DESeq2 (version 1.34.0) [105]. Beta diversity was analyzed with a PERMANOVA via the ADONIS function within the vegan R package (version 2.5–7). All figures were generated with GraphPad Prism (version 8.0) (GraphPad Software, La Jolla, CA, United States). The map in Fig. 1 was generated with the ggplot2 and sf R packages [112, 113].

## Supplementary Information

**Additional file 1.** Supplementary Figures.

**Additional file 2. Table S1**. List of each sample collected and associated metadata.

**Additional file 3. Table S2**. Differentially abundant genera between summer flounder populations collected at Fox Island and Whale Rock, determined using DESeq2.

**Additional file 4. Table S3**. Count table of resistance genes and resistance classes identified in each sample using DeepARG.

**Additional file 5. Table S4**. Counts table of chondroitin associated CAZymes (PL8, PL8_2, and GH88) identified in the metagenomically assembled genomes.

## Data Availability

The sequencing data generated for this study can be found in the NCBI Sequence Read Archive.
