# Supplementary material for: Trophic level and proteobacteria abundance drive antibiotic resistance levels in fish from coastal New England
Source: Anim Microbiome. 2023 Mar 6;5:16. doi: 10.1186/s42523-023-00236-w (PMC9990352; doi:10.1186/s42523-023-00236-w)
Supplement: Supplementary file 1 — Additional file 1. Supplementary Figures. [file 42523_2023_236_MOESM1_ESM.docx]

Supplementary Figures and Tables

Figure S1. Intra Species Microbiota Differences Between the Inner and Upper Bay Locations

Genus level relative abundance (A) and Principal coordinate analysis of Bray-Curtis Dissimilarity of Fox Island fish samples collected in 2021 (B). Genus level relative abundance (C) and Principal coordinate analysis of Bray-Curtis Dissimilarity of Whale Rock fish samples collected in 2021 (D).


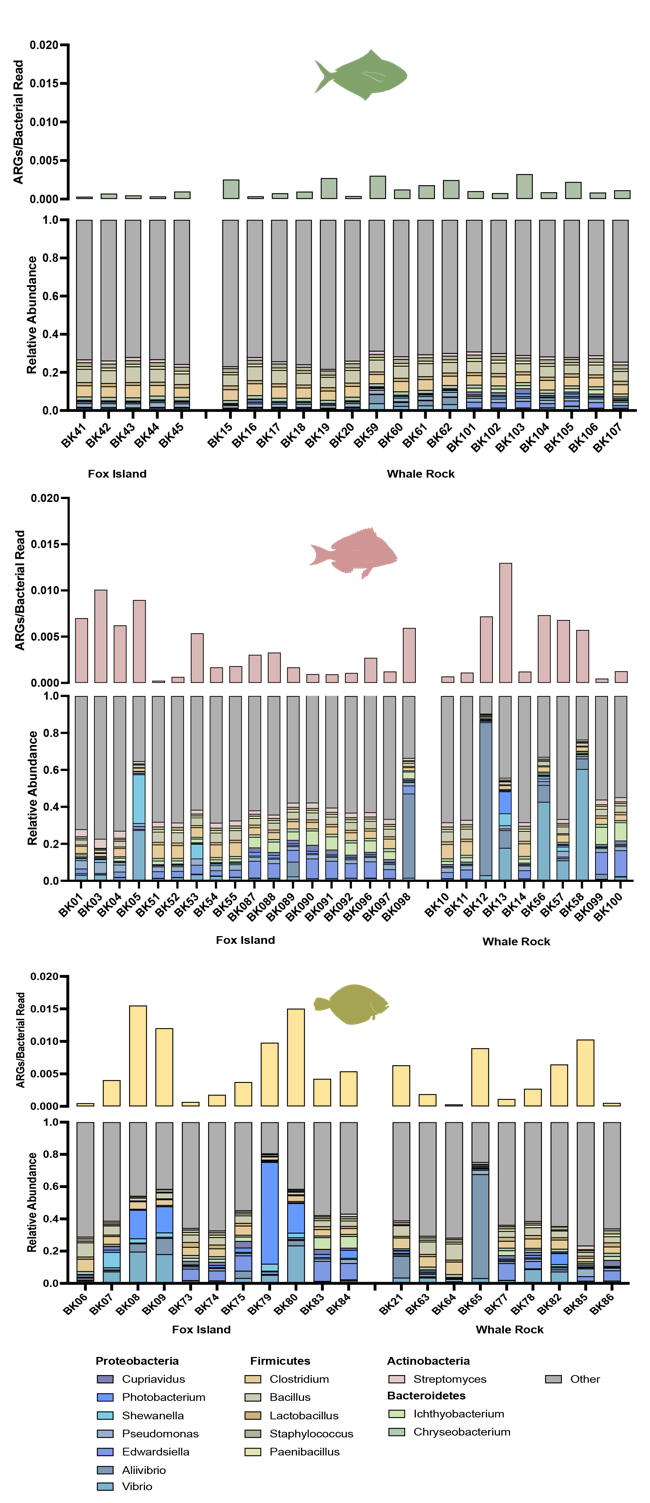


Figure S2. Heterogeneity in Proteobacteria Blooms Associated with ARG Abundance

Abundance of ARGs normalized to bacterial reads plotted for each sample separated by species. Below the normalized ARG abundance for each species is the genus taxonomy plot for the corresponding sample.

Figure S3. ARG Abundance and Association between ARGs and Proteobacteria (All Samples)

ARGs normalized to bacterial reads in each species and water samples with bars representing mean + standard error of the mean (A). Correlation between ARGs (y-axis) and Proteobacteria relative abundance of all fish/shark samples (x-axis) (r = 0.6484, R^2^ = 0.4204, p < 0.0001) (B).

Figure S4. Phylogenic Tree of Assembled Bins

Phylogenetic tree of MAGs generated using PhyloPhlAn 3.0 with nodes displaying the lowest identified taxonomy assigned by Bin Annotation Tool (BAT). The color of the node labels corresponds to the sample from which they originated – blue: water, green: butterfish, red: scup, yellow: summer flounder, grey: smooth dogfish, purple: shark.

Figure S5. Neighbor Joining Tree of *Clistobothrium montaukensis* COI Sequence

Neighbor joining tree of *Clistobothrium montaukensis* COI sequence from mako shark sample BK28 (labeled in red) with other flatworm COI sequences from NCBI, generated with NCBI Blast Tree Widget.

Table S1. List of each sample collected and associated metadata.

Table S2. Differentially abundant genera between summer flounder populations collected at Fox Island and Whale Rock, determined using DESeq2.

Table S3. Count table of resistance genes and resistance classes identified in each sample using DeepARG.

Table S4. Counts table of chondroitin associated CAZymes (PL8, PL8_2, and GH88) identified in the metagenomically assembled genomes.
